# Do Visual and Vestibular Inputs Compensate for Somatosensory Loss in the Perception of Spatial Orientation? Insights from a Deafferented Patient

**DOI:** 10.3389/fnhum.2016.00181

**Published:** 2016-04-28

**Authors:** Lionel Bringoux, Cécile Scotto Di Cesare, Liliane Borel, Thomas Macaluso, Fabrice R. Sarlegna

**Affiliations:** ^1^Aix-Marseille Université, CNRS, ISM UMR 7287Marseille, France; ^2^Cognitive Neuroscience Department and Cognitive Interaction Technology, Center of Excellence, Bielefeld UniversityBielefeld, Germany; ^3^CNRS, LNIA UMR 7260, Aix-Marseille UniversitéMarseille, France

**Keywords:** spatial orientation, body tilt, multisensory integration, deafferented patient

## Abstract

The present study aimed at investigating the consequences of a massive loss of somatosensory inputs on the perception of spatial orientation. The occurrence of possible compensatory processes for external (i.e., object) orientation perception and self-orientation perception was examined by manipulating visual and/or vestibular cues. To that aim, we compared perceptual responses of a deafferented patient (GL) with respect to age-matched Controls in two tasks involving gravity-related judgments. In the first task, subjects had to align a visual rod with the gravitational vertical (i.e., Subjective Visual Vertical: SVV) when facing a tilted visual frame in a classic Rod-and-Frame Test. In the second task, subjects had to report whether they felt tilted when facing different visuo-postural conditions which consisted in very slow pitch tilts of the body and/or visual surroundings away from vertical. Results showed that, much more than Controls, the deafferented patient was fully dependent on spatial cues issued from the visual frame when judging the SVV. On the other hand, the deafferented patient did not rely at all on visual cues for self-tilt detection. Moreover, the patient never reported any sensation of tilt up to 18° contrary to Controls, hence showing that she did not rely on vestibular (i.e., otoliths) signals for the detection of very slow body tilts either. Overall, this study demonstrates that a massive somatosensory deficit substantially impairs the perception of spatial orientation, and that the use of the remaining sensory inputs available to a deafferented patient differs regarding whether the judgment concerns external vs. self-orientation.

## Introduction

The perception of spatial orientation relies on the central processing of multisensory information such as vestibular, visual and somatosensory inputs (MacNeilage et al., 2008 for a review) and prior knowledge about gravity (Lacquaniti et al., 2014). The contribution of sensory signals to spatial perception has been notably addressed by studying the effect of sensory deficits. On the one hand, impairments in spatial abilities have been observed even years after the deficit occurrence (e.g., Foster, 1994). On the other hand, remarkable compensatory mechanisms have been shown in sensory-impaired patients, allowing them to preserve or even enhance spatial perception (Lessard et al., 1998; Van Boven et al., 2000; Bavelier et al., 2006). Such sensory compensation, based on the unimpaired sensory inputs, seems to depend on the type of sensory deficit, environmental properties and task requirements (Lacour et al., 2009; Cousins et al., 2014). Here, we investigated how a massive loss of somatosensory inputs may impact spatial perception by studying the ability of a deafferented patient to use some remaining visual and vestibular cues in two distinct spatial orientation tasks involving external vs. self-orientation judgments.

A large amount of studies which investigated how sensory impairments could influence spatial perception dealt with the impact of visual deficits (Pasqualotto and Proulx, 2012 for a review). Considering the critical implication of vision for spatial orientation (Howard, 1982), one may expect that visual impairment could bias the perceived orientation of objects and/or the body. While some studies indeed reported degraded spatial abilities in congenitally-blind subjects (e.g., Seemungal et al., 2007), some others have shown similar or even improved spatial performance in blind subjects as compared to subjects without visual deficit. For instance, the haptic perception of objects orientation did not differ between blind and blindfolded sighted subjects (Gentaz and Hatwell, 1998). Furthermore, the perception of self-motion direction was found more accurate in congenitally blind with respect to blindfolded sighted subjects (Moser et al., 2015). Presumably in these conditions, vestibular and somatosensory signals may have compensated for the absence of vision.

When considering the influence of vestibular deficits on the perception of spatial orientation, it has been shown that unilateral vestibular loss yields detrimental effects on the adjustment of a visual rod to gravitational vertical (i.e., Subjective Visual Vertical or SVV, Tabak et al., 1997; Lopez et al., 2007) and on the perception of body orientation (Aoki et al., 1999). However, bilateral vestibular loss does not result in such significant impairments (Bisdorff et al., 1996; Ito and Gresty, 1996; Anastasopoulos et al., 1999; Bringoux et al., 2002) In this case, compensatory processes, mainly based on vision (Bronstein et al., 1996; Guerraz et al., 2001; Lopez et al., 2007) and possibly on somatosensory signals, may also account for the preserved perception of spatial orientation.

Less is known regarding the consequences of somatosensory loss on the perception of spatial orientation, although its influence has been extensively studied in the context of motor control (Rothwell et al., 1982; Sanes et al., 1984; Sainburg et al., 1993; Sarlegna et al., 2010). Stroke patients suffering single-hemisphere somatosensory lesions exhibit a substantial bias during SVV adjustments toward the hypoesthetic side (Anastasopoulos et al., 1999) and adjustments are also more variable (Barra et al., 2010; Saeys et al., 2012). On the other hand, only severe hemihypoesthesia biases the subjective postural vertical (SPV), that is the alignment of whole-body orientation with the gravitational vertical (Anastasopoulos et al., 1999). In the same vein, a peripheral and symmetric somatosensory loss did not prevent the control of sitting posture without back-support (Blouin et al., 2007) suggesting that vestibulo-spinal pathways remain sufficient to control body posture without touch and proprioception.

The purpose of the present study was to assess the perception of spatial orientation in a rare case of massive yet selective somatosensory deafferentation. Such a sensory deficit raises the issue of compensatory mechanisms when only visual or vestibular inputs remain available for spatial perception. In line with recent models of multisensory integration, the remaining cues following sensory deficit should be reweighted according to their noise properties and processed with priors that the patients may have built with experience and perceptual expectancies (Vingerhoets et al., 2009; Clemens et al., 2011). In the present study, a well-characterized deafferented patient (GL; Forget and Lamarre, 1987; Blouin et al., 1995; Sarlegna et al., 2010) was compared to age-matched subjects in two spatial orientation tasks involving gravity-related judgments. First, the perception of object/external orientation was addressed through a SVV task in which participants had to align a rod with the gravitational vertical while visual surroundings could be tilted (i.e., portable Rod-and-frame Test [RFT], Oltman, 1968). Second, the perception of body/self orientation was investigated through a self-tilt detection task in which participants had to judge whether they felt tilted forward from vertical while facing different visuo-postural conditions, as in our previous work on healthy young participants (Scotto Di Cesare et al., 2014). Comparing the results in these two experimental tasks enabled us to investigate whether compensatory processes following somatosensory loss, if any, could generalize to different spatial perception tasks. Overall, we expected that the patient with a massive somatosensory loss would exhibit a greater reliance on visual and/or vestibular cues compared to Controls.

## Materials and methods

### Participants

One 65-year-old somatosensory-deafferented patient and 8 healthy, age-matched “Controls” (5 females and 3 males; mean age ± SD: 65.2 ± 4.6 years) participated in this study. All the subjects were naive to the specific purpose of the experiment, which was approved by the institutional review board of the Institute of Movement Sciences. They gave their informed consent prior to the study, in accordance with the ethical standards set out in the 1964 Declaration of Helsinki. Participants were self-declared right-handed and had normal or corrected-to-normal vision. Stereoscopic vision was checked using the Randot Stereotest®, with all individual scores greater than 70 s of arc. None of the Controls had any relevant medical history as no neurological or sensorimotor disorder was reported. The deafferented patient, known as GL, had two severe episodes of extensive polyneuropathy (at the ages of 27 and 31 years) affecting her whole body below the nose (for detailed descriptions, see Forget and Lamarre, 1987; Cole and Paillard, 1995; Sarlegna et al., 2010). Clinical tests revealed a specific loss of large-diameter, myelinated Aβ afferents which resulted in a complete loss of touch, vibration, pressure, tendon reflexes, and sense of movement and position in the four limbs, the trunk being moderately affected. Tests carried out in the ENT Department (Clairval, Marseille) showed that her vestibular function is preserved, in accordance with previous reports (Cole and Paillard, 1995; Guillaud et al., 2011). Similarly, according to tests performed in the Ophthalmology Department (La Timone, Marseille), no deficit in visual acuity and perimetry was found.

### Experimental setup

#### Apparatus for the rod-and-frame test (RFT)

**Figure 1:**
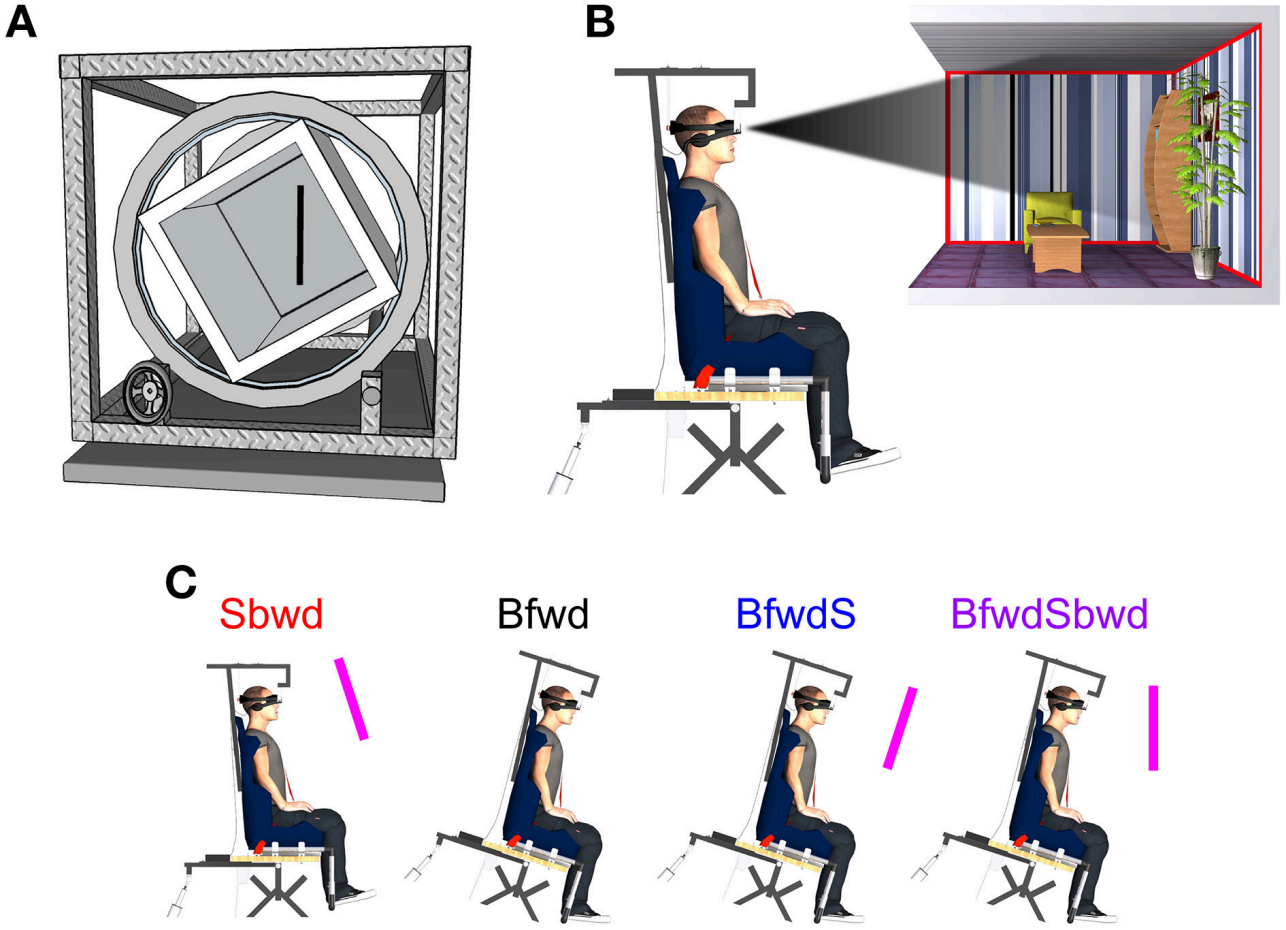
**(A)** Illustration of the portable rod-and-frame apparatus (RFT) replicated from Oltman (1968). **(B)** The tilting chair and the visual scene projected in the Head-Mounted Display. **(C)** Experimental conditions manipulated in the self-tilt detection session. ***Sbwd***: backward rotation of the visual scene (top toward the subject) without body rotation; ***Bfwd***: forward rotation of the body without any visual scene; ***BfwdS***: forward body rotation with a visual scene remaining static relative to the subject; ***BfwdSbwd***: forward body rotation with backward visual scene rotation relative to the subject.

In the first part of the experiment (RFT session), we used a replication of the portable RFT apparatus developed by Oltman (1968) to measure SVV estimates while visual surroundings were tilted. The device was composed of a box (57 cm deep × 31 cm wide × 31 cm high) made of translucent white surfaces whose inside edges and corners were marked by black lines (Figure 1A). Subjects were seated upright so that their face was aligned with the front, open edge of the box (the center of the box corresponding to their straight ahead). From this open edge, subjects could not see the outer environment and could only see inside the box, in particular the black square frame and a black rod at the opposite end (apparent size of the square: ~30.5°). The bright interior of the box was shadowless and no orientation cues from external lighting were available. The whole box, and thus the frame, could be tilted by the experimenter at different roll orientations. A black rod (1 cm large and 30 cm long; apparent length: ~29.5°) could be rotated around the center of the frame by the subject or by the experimenter via independent hand-levers. A protractor, fixed on a disc mounted at the rear of the box and visible only to the experimenter, indicated the deviation of the frame and the rod from gravitational vertical. Subjects' eye level coincided with the axis of rotation of the rod and the frame. Their head was stabilized with a chin-rest and a head-rest.

#### Tilting chair and virtual reality head-mounted display

In the second part of the experiment (Self-tilt detection session), subjects were seated and firmly attached to a tilting chair with a 6-point seatbelt. The tilting chair could be rotated in pitch, around an axis positioned under the seat (Figure 1B). The rotation was produced by lengthening/shortening an electric jack (Phoenix Mecano®, thrust: 3 kN, clearance: 0.6 m, precision 0.12 mm) attached to the back of the seat. The angular profile of the tilt was servo-assisted using an inclinometer fixed to the chair (AccuStar®; resolution: 0.1°; range: ±60°). Throughout the trials, earphones provided white noise to mask any auditory cues. Two push buttons (one per hand) were used to record subjects' response about self-tilt perception.

A 3D head-mounted display (HMD, 3D Cybermind hi-Res9001®; resolution: 800 × 600 pixels; field of view: 31.2° diagonal for each eye) was fixed onto a headrest attached to the seat. This headrest was adjustable in height to the subjects' size. The HMD was used to display a stereoscopic 3D visual scene composed of a fully furnished and polarized room (3 m wide × 6 m long × 2.25 m high, Figure 1B). The distance of the virtual scene front from subjects' eye in the transverse plane was set at 1.7 m, such that it remained fully visible according to the HMD field of view. The virtual scene could rotate in pitch around the same axis as the rotating chair. The HMD device prevented subjects from viewing the experimental setup and their actual body configuration with respect to the external space. A real-time acquisition system (ADwin-Pro®) running at 10 kHz and a customized software (Docometre®) were used to synchronously record subjects' responses and control the HMD visual background and/or chair rotations.

### Procedure

#### RFT session

Participants were first asked to perform SVV judgments in a classic RFT procedure. Specifically, they had to align a tilted rod along the gravitational vertical when facing a visual frame. The frame orientation was modified according to a standardized order (Isableu et al., 1997; Bringoux et al., 2009; Scotto Di Cesare et al., 2015): 0°, −18°, +18°, −8°, +8°, −28°, +28°, −38°, +38°, 0° positive values indicate rightward tilts while negative values indicate leftward tilts relative to the gravitational vertical). For each of the 9 frame orientations, 2 trials were performed with initial rod orientations alternatively set right or left at a magnitude ranging from ±10° to ±50° relative to vertical.

Prior to the session, instructions were given to the subjects: special attention was given to the definition of “gravitational vertical” using verbal explanations and sketches. Subjects were required to keep their eyes closed throughout the session except when they were explicitly asked to align the rod with the gravitational vertical. To that end, Controls rotated a hand-lever allowing them to adjust rod orientation with their unseen left hand, while GL, who was unable to manipulate the hand-lever without vision, gave verbal instructions to the experimenter for setting the rod at the desired orientation (e.g., further left, or further right). Pre-tests confirmed that both response modes yielded similar results on SVV settings. When participants were satisfied about their judgment, they verbally informed the experimenter and closed their eyes until the next trial. Overall, this RFT session comprised 36 trials and lasted 20 min on average. It was repeated twice for GL, before and after the self-tilt detection session, to verify responses' consistency, which is well established in healthy subjects (Bergman, 1979).

#### Self-tilt detection session

In this second experimental session, participants, seating on the tilting chair, were asked to indicate whether they felt being tilted forward (that is, away from the gravitational vertical). Specifically, they were forced to either answer: “Yes (I feel tilted forward)” or “No (I do not feel tilted forward).” Controls had to respond either with the left or right hand-held push button, which corresponded to Yes and No respectively, while GL, who could not use push-buttons with her unseen hands, was instructed to verbally give her response “Yes” or “No.”

Each trial began with the chair and the visual scene aligned with the gravitational vertical (i.e., upright). The chair and/or the visual scene rotated up to 18° at 0.05°s^−1^ following a 10 s acceleration phase at 0.005°s^−2^, below the threshold for semicircular canals stimulation (Benson, 1990). Subjective responses were prompted by an auditory tone at each degree of tilt, i.e., every 20 s. At the end of the trial, participants were asked to close their eyes, and the chair was brought back to vertical if it actually had moved. The chair rotation back to vertical varied in terms of kinematics, so that the subjects could not infer the angle of tilt previously reached. Between trials, the HMD was removed and a period of rest in full ambient light, during at least 1 min, was consistently provided before the next trial started. This resting period was used to suppress post-rotational effects and to limit possible fatigue.

In this self-tilt detection session, four experimental conditions were presented (Figure 1C; see also Scotto Di Cesare et al., 2015): ***Sbwd***: backward rotation of the visual scene (top toward the subject) without body rotation; ***Bfwd***: forward rotation of the body without any visual scene; ***BfwdS***: forward body rotation with a visual scene remaining static relative to the subject; ***BfwdSbwd***: forward body rotation with backward visual scene rotation relative to the subject. All subjects performed 3 trials in each of the 4 aforementioned conditions, which were presented in a pseudo-random, counterbalanced order, to avoid any potential learning effect. A training phase without body and/or visual scene rotation was provided before the data collection actually started, to familiarize subjects with the task. Two catch trials without effective body and scene tilt were randomly inserted in the session, to further assess the reliability of subjects' estimates. The whole experimental session lasted about 2 h.

### Data analysis

From the RFT session, we analyzed the SVV measures for each frame orientation and computed the mean RFT score for each subject according to the classic Nyborg and Isaksen's method (Nyborg and Isaksen, 1974), following the equation:

$$\begin{array}{l} RFTscore=\sum \text{E}rr\left(R\right)∕R-\sum \text{E}rr\left(T\right)∕T \end{array}$$

“E*rr*(*R*)” is the signed error recorded when the frame was tilted 18° rightward; “*R*” is the number of rightward frame tilts; “E*rr*(*T*)” is the mean signed error recorded at 18° of both rightward and leftward frame tilts; “*T*” is the total number of rightward and leftward frame tilts.

**Figure 2:**
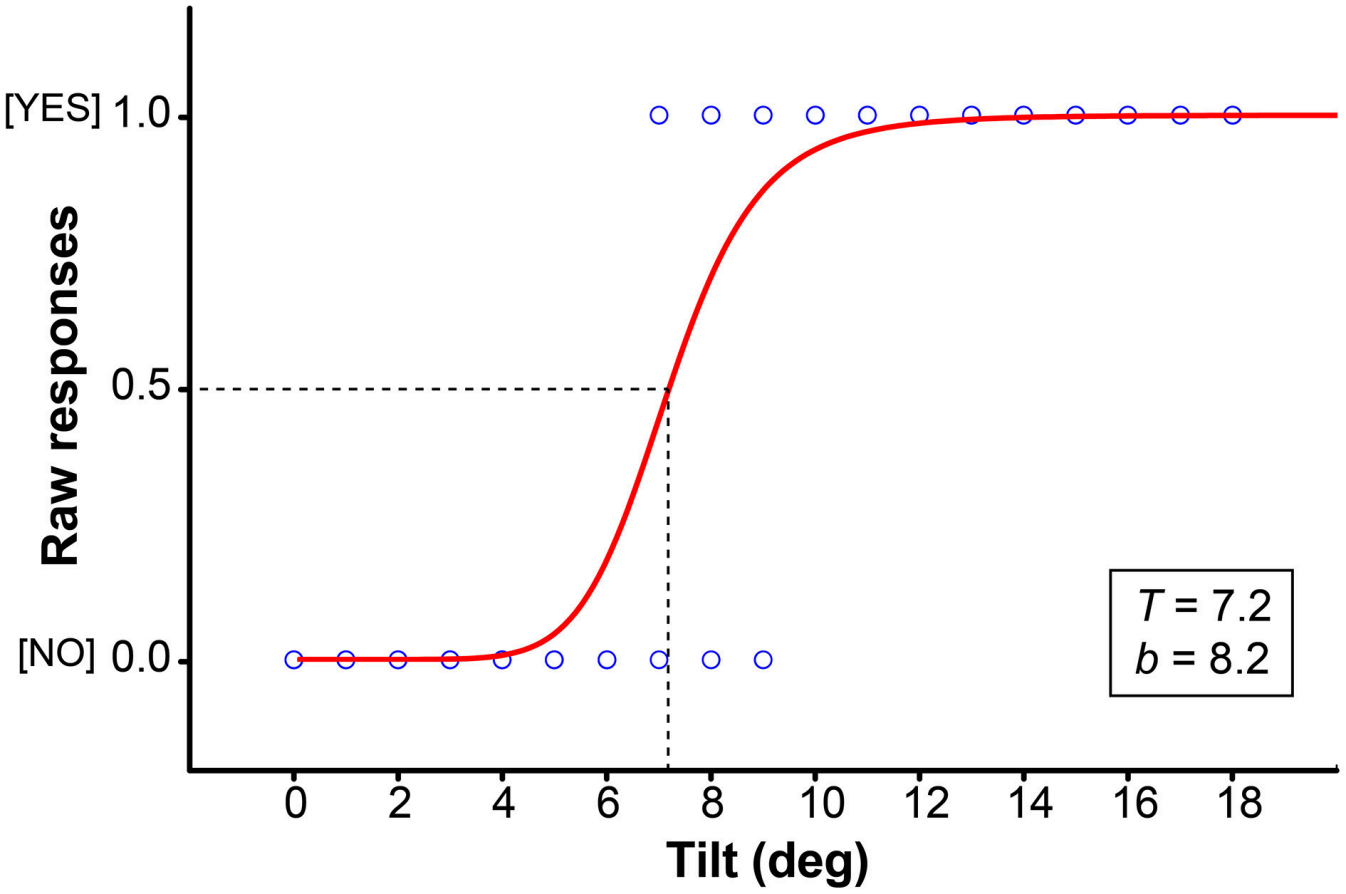
**Representative psychometric function defined for a Control subject in a typical trial (e.g., ***Bfwd*** condition)**. A non-linear regression (Probit) was applied to the raw judgments to define a threshold for self-tilt detection.

From the Self-tilt detection session, individual thresholds were determined for each experimental condition. Responses were converted into binary values, with “1” corresponding to the response “Yes, I feel being tilted forward” and “0” to the response “No, I do not feel being tilted forward.” A Probit model, using a non-linear regression analysis for binomial values (least square fitting) was applied to the data in order to determine the threshold corresponding to 50% of probability of expressing the feeling of being tilted (Figure 2).

The Probit function was defined as follows:

$$\begin{array}{l} Pi=\frac{1}{1+{\left(\frac{At}{T}\right)}^{b}} \end{array}$$

“*P*” is the probability in expressing the feeling of being tilted for a given condition “*I*.” “*At*” corresponds to the angle of tilt during this condition and “*T*” to the self-tilt detection threshold for this condition (i.e., angle of tilt for *P* = 0.5). “*b*” is the slope of the tangent of the curve at its inflection point and constitutes an estimate of the discrimination sensitivity relative to the chosen increments.

To compare GL's data to those of Controls in the RFT session, *t*-test comparisons of a single value to a population sample were used (Sarlegna et al., 2010). In the self-tilt detection session, mean data of Controls across conditions were compared using a one-way repeated measures analysis of variance (ANOVA) and appropriate *post-hoc* comparisons (i.e., Newman-Keuls tests) since data respected normality assumption (i.e., Lilliefors tests). In this session, GL performance was evaluated with respect to the mean ±95% confidence intervals characterizing Controls' performance in each condition. For all tests, the significance threshold was set at 0.05.

## Results

### Rod-and-frame test (RFT)

**Figure 3:**
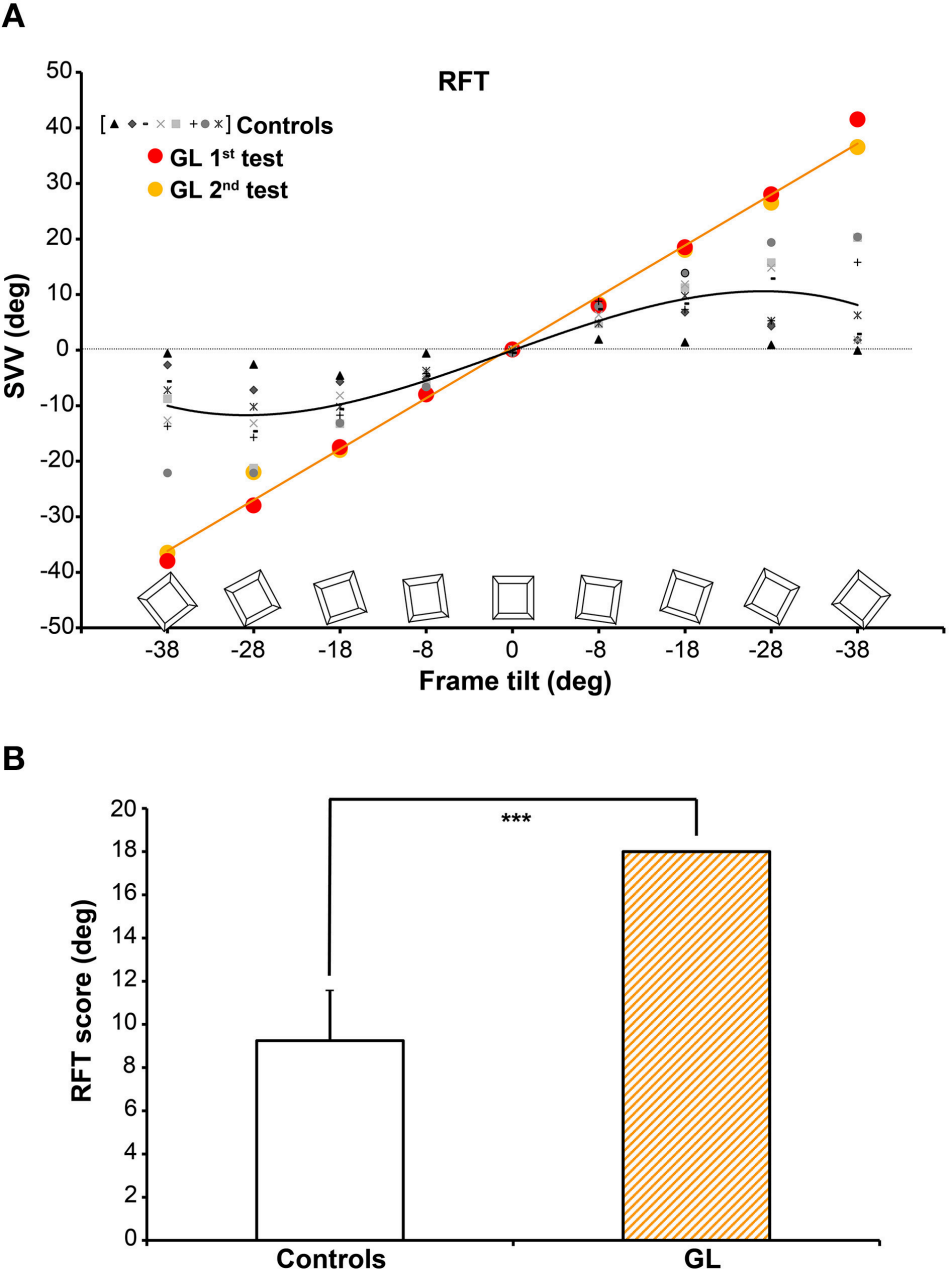
**(A)** SVV settings as a function of frame tilt in the RFT session for Controls. Each symbol represents a given subject. While SVV classic sinusoidal modulations as a function of frame tilt magnitude were observed for Controls (*y* = −0.0003x^3^ − 0.0006x^2^ + 0.62x − 0.08; R^2^ = 0.99), SVV estimates corresponded ~100% to the frame orientation for the deafferented patient (*y* = 0.98x + 0.52; R^2^ = 1.00). **(B)** Mean RFT scores obtained for Controls and the deafferented patient GL. Since 0° score corresponds to extreme visual field independence and 18° score corresponds to extreme visual field dependence, this figure shows that the deafferented patient is entirely field dependent according to RFT classification. Error bars for Controls data represent 95% confidence interval. ^***^p < 0.001.

Figure 3A illustrates the mean SVV settings (i.e., mean signed errors in rod adjustment relative to gravitational vertical) for the deafferented patient GL and age-matched Controls as a function of frame tilt. Beyond the usual between-subjects variability, Controls exhibited typical sinusoidal SVV profiles characterizing a classic “frame effect” (e.g., Bringoux et al., 2009). Their maximal SVV deviations away from the vertical, and thus toward the tilted frame, were recorded for ±18° or ±28° of tilt, while their adjustments tended to become closer to vertical for smaller (i.e., ± 8°) or larger frame tilts (i.e., ±38°, close to a diagonal frame). Both Controls and GL were particularly accurate for SVV settings when facing a non-tilted frame (i.e., less than 1° of error on average for a 0° frame orientation). One of the main findings of the present study is that GL estimates drastically differed from those of Controls when the frame was tilted (See Table 1 for statistical comparisons between GL and Controls at each frame tilt magnitude). Her SVV adjustments were almost systematically equal to the angle of frame tilt (see video in Supplementary Materials), even at the largest tilts (i.e., ±38°). This was observed in both RFTs performed by GL before and after the self-tilt detection session (Figure 3A).

**Table 1:** **Rod and Frame Test**.

**Controls**			**GL**						
**Frame tilt**	***X***	**+/−95% CI**	*X* **1st Test**		**Statistical comparison**		***X* 2nd Test**	**Statistical comparison**	
					***t***	***p***		***t***	***p***
−38°	−9.2	[−15.0/−3.4]	−38.0		11.82	<0.001	−36.5	11.20	<0.001
−28°	−13.4	[−18.9/−7.8]	−28.0		6.21	<0.001	−22	3.66	<0.01
−18°	−9.7	[−12.4/−7.0]	−17.5		6.79	<0.001	−18	7.22	<0.001
−8°	−4.4	[−6.0/−2.9]	−8		5.42	<0.001	−8	5.42	<0.001
0°	−0.1	[−0.4/0.2]	−0.25		1.18	ns	0.0	−1.00	ns
8°	5.8	[4.0/7.7]	8.0		−2.80	<0.05	8.25	−3.12	<0.05
18°	8.8	[5.6/12.0]	18.5		−7.15	<0.001	18	−6.78	<0.001
28°	9.8	[4.2/15.4]	28.0		−7.71	<0.001	26.5	−7.07	<0.001
38°	8.6	[1.3/15.9]	41.5		−10.67	<0.001	36.5	−9.05	<0.001

*Statistical report for mean Subjective Vertical comparisons between Controls and GL estimates at each frame tilt*.

A further quantitative analysis of the frame effect revealed that despite the high RFT score obtained for Controls (see Scotto Di Cesare et al., 2015 for a comparison with younger adults), GL score, averaged across the two repetitions, was even higher than that of Controls [*t*_(8)_ = −7.49; *p* < 0.001]. GL score was indeed largely beyond the 95% confidence interval of the Controls score, as illustrated in Figure 3B. GL score (18°) actually corresponded to the orientation of the visual frame, meaning that she exhibited full visual field dependence for the perception of object orientation.

### Self-tilt detection threshold

Overall, although the task was considered difficult by the subjects, the perceptual transitions in the feeling of being tilted -when existing- appeared relatively suddenly in almost all trials. Furthermore, subjects never reported any self-tilt sensation during the catch trials, suggesting that they were compliant with the task requirements.

Among all the trials carried out by Controls, few of them led to no self-tilt sensation (***Sbwd***: 8/24: ***Bfwd***: 0/24; ***BfwdS***: 2/24; ***BfwdSbwd***: 1/24). The consistency of the self-tilt detection sensitivity across conditions was analyzed for Controls using a 4-condition repeated-measures ANOVA on the “b” values of the Probit functions. This analysis did not reveal any significant difference between the discrimination sensitivity across conditions (“b” *[Mean* ±*SD]* = 6.3 ± 3.5; *F*_(3, 12)_ = 1.03; *p* = 0.42).

**Figure 4:**
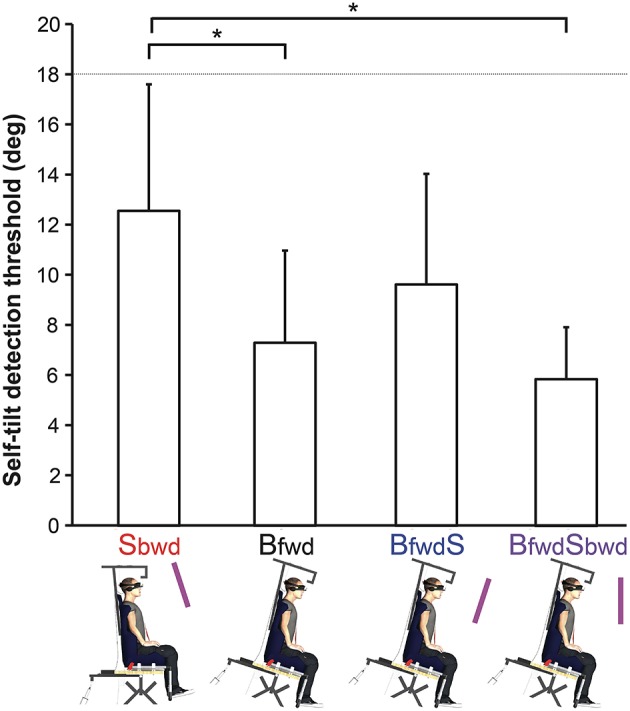
**Mean self-tilt detection thresholds as a function of experimental conditions for controls**. The deafferented patient never felt any tilt up to the largest tilt angle (dashed line) across the different trials in all the conditions she was exposed to. Error bars represent 95% confidence intervals. ^*^p < 0.05.

The ANOVA on the mean self-tilt detection thresholds for Controls revealed a significant effect of experimental conditions [*F*_(3, 21)_ = 3.43; *p* < 0.05; Figure 4]. *Post-hoc* analysis revealed that the threshold was significantly higher when the visual scene alone was rotated (***Sbwd*** = 12.6°), as compared to when the body alone was rotated (***Bfwd*** = 7.3°; *p* < 0.05) or when both the body and the visual scene were rotated (***BfwdSbwd*** = 5.9°; *p* < 0.05).

Most importantly and as a core result, GL' self-tilt detection threshold differed from that of Controls in all conditions, since that the patient never reported any self-tilt sensation, in any trial of any condition (see video in Supplementary Materials). The maximal angle of tilt we manipulated (18°), up to which GL never felt tilted, was out of the 95% Confidence Intervals calculated on the threshold values for Controls in the 4 experimental conditions (Figure 4). In contrast to Controls, visual scene manipulation had no influence on GL self-tilt detection and none of the -extremely slow- physical tilt up to 18° (i.e., ***Bfwd***, ***BfwdS***, ***BfwdSbwd***) evoked a self-tilt sensation for the deafferented patient.

## Discussion

The present study aimed at investigating whether a massive loss of somatosensory inputs may drastically change the perception of spatial orientation. By testing external (object) orientation and self-orientation perception of a deafferented patient (GL), we expected to highlight compensatory processes based on visual or vestibular inputs. GL's perception of external orientation, investigated by SVV judgments in a RFT, was found to strikingly depend on visual inputs. However, for self-orientation perception, GL never felt being tilted during slow tilts of the visual scene, her body or a combination of both up to 18°, contrary to healthy Controls who were able to detect changes in self-orientation relative to vertical. Overall this study demonstrates that critical somatosensory deficit yields substantial impairments in spatial orientation perception, and that the use of the remaining sensory inputs available to a deafferented patient differs regarding whether the estimate concerns external vs. self-orientation. The implication of these findings will be discussed in the following sections.

### Self- vs. external orientation perception: Different contributions of vision

For external orientation perception, the deafferented patient GL seemed to exclusively refer to vision, as her SVV estimates were fully biased by the frame tilts. Between +38° and −38° of tilt, mean responses from the patient almost differed by 80° (contrary to Controls who tended to better align their SV with gravity at these large angles). It is possible that the systematic succession of left and right presentations of frame tilt led to strong expectations relative to the main direction of the -anchoring- visual reference for GL. In addition, the slight but existing asymmetry between the orientation of the frame sides (bottom/up vs. left/right) relative to the longitudinal body axis at ±38°, may have also helped GL determine the main visual axis of reference for orientation (i.e., the closest lines of the frame relative to her idiotropic axis constituting the directional reference for her judgments). Overall, the key finding in the RFT session is that GL completely relies on the main orientation of the visual frame to perform her SVV estimates, contrary to Controls.

This is in line with previous work highlighting the reliance of deafferented patients on vision for motor control (Rothwell et al., 1982; Blouin et al., 1993; Sarlegna et al., 2010). It is also coherent with other findings which reported greater visual field dependence in people suffering from Parkinson's disease, also known to alter somatosensory processing (Azulay et al., 2002). The prominent role of vision in the perception of spatial orientation is well established. Pioneer studies (Asch and Witkin, 1948; Witkin and Asch, 1948) first attempted to quantify this visual influence upon the perception of object orientation by asking observers to adjust a rod surrounded by a tilted frame at the gravitational vertical (i.e., RFT). While the orientation of the visual frame consistently influences some subjects (i.e., field-dependent subjects, FD), others remain relatively immune to visual cues (i.e., field-independent subjects, FI). According to a general assumption, FD subjects are supposed to rely less on gravity-related vestibular and/or somatosensory inputs, as compared to FI subjects (Isableu et al., 2010). In line with this idea, the fact that the deafferented patient fully relied on visual cues for setting a rod to the vertical suggests that she may not use gravity-related vestibular cues for the perception of external orientation.

Surprisingly, in the self-tilt detection task, GL did not use visual orientation cues from the surroundings. This shows that visual dependence, defined on the basis of SVV judgments in a RFT session, does not necessarily extend to a self-orientation perception task. This is consistent with previous studies which investigated the generalization of field dependence across spatial perceptual abilities and demonstrated that the classification of FD/FI subjects based on RFT may be invalid in other spatial judgments (Barnett-Cowan et al., 2010; Scotto Di Cesare et al., 2015). Indeed, perceptual upright estimates when tilted (Barnett-Cowan et al., 2010) or self-tilt detection involving slow changes in postural cues (Scotto Di Cesare et al., 2015) were not significantly related to FD/FI categorization issued from RFT. Overall, these data highlight a clear dissociation between self-orientation perception and external orientation perception. This gives additional support to the view that judging postural orientation and judging object orientation rely on distinct sensory integration processes (Bronstein, 1999).

### Are gravity-related vestibular cues of any help for orientation perception?

The present findings showed that visual orientation cues were not used by the deafferented patient for self-orientation perception. Based on current theories on cross-modal plasticity (Auvray and Harris, 2014), one could expect that vestibular cues could take over the lack of visual contribution in the self-tilt detection task. Surprisingly, the results of the deafferented patient did not confirm a greater use of vestibular inputs for self-orientation perception compared to healthy controls. Although the very slow body tilts prevented any motion sensation issued from the semi-circular canals (Benson, 1990), the otoliths, usually presented as gravity-sensitive organs (Goldberg and Fernandez, 1984), remained susceptible to convey informative cues about whole-body orientation. That GL is able to use vestibular system has been shown in previous studies investigating sensorimotor processes (vestibulo-ocular: Blouin et al., 1995; vestibulospinal: Blouin et al., 2007). Therefore, the fact that GL was never able to detect self-tilt up to 18° may mainly result from her inefficiency to calibrate changes in vestibular inputs at a perceptual level. Similar conclusions have been drawn by Blouin et al. (1995) who investigated magnitude estimates of passive whole-body rotations in yaw with the same deafferented patient. The inefficiency of using vestibular inputs for slow self-tilt detection may not be specific to a deafferented patient however, since healthy subjects embedded in a full body cast were also greatly impaired in perceiving very slow body tilts on the sole basis of their remaining otolith inputs (Bringoux et al., 2003). Overall, our findings strongly suggest that isolated otolith signals cannot be considered as an accurate source of graviception at a perceptual level.

Previous findings have stressed that otolith sensitivity to dynamic stimuli is greater than to static ones, outlining the importance of the rate of change of the vestibular afferent information (Gianna et al., 1996). Also, the phase response of the vestibular neurons in the brain stem (Angelaki et al., 2004) or in the parieto-insular vestibular cortex (Chen et al., 2010) has been reported to span from jerk to velocity (i.e., no signal of position). Furthermore, the vestibular system rarely works alone (Barnett-Cowan, 2013) and may require other sensory signals to be interpreted at a perceptual level. In line with this idea, primary as well as secondary afferent vestibular projections are known to merge with other sensory inputs in several brain areas (e.g., vestibular nuclei, Jamali et al., 2014; Parieto-Insular Vestibular Cortex, Lopez and Blanke, 2011) to solve stimulus ambiguity and help signal interpretation. Additional studies are thus necessary to test the hypothesis that the deafferented patient may use vestibular inputs mostly as a trigger for motion perception, when the change in the stimulus is highly noticeable and possibly requires fast decisional reactions.

### Sensory compensation vs. reference frame selection

The extreme visual dependence observed for the deafferented patient GL in the RFT session may illustrate the prominent role of vision for compensating somatosensory loss when judging the orientation of external objects. Alternatively, this may also reflect the exclusive use of an allocentric frame of reference (i.e., here relative to visual surroundings) for coding the orientation of objects (Howard, 1982; Bringoux et al., 2009; see also Blouin et al., 1993). In the self-tilt detection session however, both visual and vestibular cues were not used by the patient, demonstrating that there was no sensory compensation at work for GL when considering self-orientation perception. It may also suggest that the task was performed neither in an allocentric nor in a geocentric (i.e., gravity-related) frame of reference.

Previous studies have already highlighted the involvement of an egocentric frame of reference for spatial orientation perception, even when the task requires the judgments to be performed relative to external references (Coleman and Durgin, 2014). For instance, estimates of the subjective horizon (i.e., the plane orthogonal to gravity passing through the eyes) were found to be linearly biased as a function of the magnitude of head or body pitch tilt (Bringoux et al., 2007; Bourrelly et al., 2014). According to the pioneer work of Mittelstaedt (1983), the longitudinal body axis could also define an “idiotropic vector” toward which vertical estimates are attracted, particularly when the body is tilted. In line with this interpretation, some more recent data support the existence of prior estimates for upright perception, based on the idiotropic vector (MacNeilage et al., 2007, 2008; De Vrijer et al., 2008; Vingerhoets et al., 2009; Clemens et al., 2011), that could become critical under unusual sensory contexts (i.e., microgravity). Here we postulate that somatosensory loss may considerably enhance the “prior for upright” as a reference for self-orientation perception. This may have led the deafferented patient to ignore slow-changing information for judging her body orientation. In other words, the absence of somatosensory inputs could increase perceptual state expectations of being upright during self-tilt detection at slow velocities.

Our data also fit with models using Bayesian rules that may operate on sensory reweighting processes and particularly with predictions based on the noise properties of the sensory modalities involved in spatial orientation (Vingerhoets et al., 2009; Clemens et al., 2011). Specifically, due to the noise level of body sensors, Clemens et al. (2011) predicted large errors in subjective body tilt and subjective visual vertical for patients with somatosensory loss, even though the otolith signal is accurate. This is exactly what we found in this study.

## Conclusion

The present findings showed that sensory compensation following somatosensory loss is present for external orientation perception, but is lacking for self-orientation perception. Clearly, the massive loss of somatosensory inputs resulted in a complete reliance on vision for the perception of object's verticality. However, visual and vestibular inputs are inefficient to provide relevant information for self-tilt detection at slow velocities. The role of sensory expectations and priors (Summerfield and de Lange, 2014) could be here critical in the reference frame selection, in particular for self-orientation perception. Yet, the way sensory integration may interact with central predictions of the actual perceptual state needs to be further investigated, notably in patients with sensory impairments.

## Author contributions

LB designed and performed experiments, analyzed data and wrote the paper; CS designed and performed experiments, and wrote the paper; LB wrote the paper; TM performed experiments; FS designed experiments and wrote the paper.

### Conflict of interest statement

The authors declare that the research was conducted in the absence of any commercial or financial relationships that could be construed as a potential conflict of interest.
